# Aging-induced dysbiosis worsens sepsis severity but is attenuated by probiotics in D-galactose-administered mice with cecal ligation and puncture model

**DOI:** 10.1371/journal.pone.0311774

**Published:** 2024-10-18

**Authors:** Chalisa Pinitchun, Wimonrat Panpetch, Thansita Bhunyakarnjanarat, Kanyarat Udompornpitak, Huy Thanh Do, Peerapat Visitchanakun, Dhammika Leshan Wannigama, Suwasin Udomkarnjananun, Monruedee Sukprasansap, Tewin Tencomnao, Pattarin Tangtanatakul, Asada Leelahavanichkul

**Affiliations:** 1 Department of Microbiology, Center of Excellence on Translational Research in Inflammation and Immunology (CETRII), Chulalongkorn University, Bangkok, Thailand; 2 Faculty of Medicine, Department of Microbiology, Chulalongkorn University, Bangkok, Thailand; 3 Faculty of Allied Health Sciences, Department of Transfusion Sciences and Clinical Microbiology, Chulalongkorn University, Bangkok, Thailand; 4 Faculty of Science, Department of Microbiology, Burapha University, Chonburi, Thailand; 5 Department of Infectious Diseases and Infection Control, Yamagata Prefectural Central Hospital, Yamagata, Japan; 6 Department of Infectious Diseases and Infection Control, Pathogen Hunter’s Research Collaborative Team, Yamagata Prefectural Central Hospital, Yamagata, Japan; 7 Yamagata Prefectural University of Health Sciences, Yamagata, Japan; 8 Faculty of Health and Medical Sciences, School of Medicine, The University of Western Australia, Perth, WA, Australia; 9 Biofilms and Antimicrobial Resistance Consortium of ODA Receiving Countries, The University of Sheffield, Sheffield, United Kingdom; 10 Faculty of Medicine, Department of Medicine, Division of Nephrology, Chulalongkorn University, Bangkok, Thailand; 11 Institute of Nutrition, Food Toxicology Unit, Mahidol University, Salaya Campus, Phutthamonthon, Na-khonpathom, Salaya, Thailand; 12 Faculty of Allied Health Sciences, Center of Excellence on Natural Products for Neuroprotection and Anti-Ageing (Neur-Age Natura), Chulalongkorn University, Bangkok, Thailand; 13 Faculty of Allied Health Sciences, Department of Clinical Chemistry, Chulalongkorn University, Bangkok, Thailand; Charotar Institute of Applied Sciences: P D Patel Institute of Applied Sciences, INDIA

## Abstract

**Introduction:**

Despite the well-established effects of aging on brain function and gut dysbiosis (an imbalance in gut microbiota), the influence of aging on sepsis-associated encephalopathy (SAE) and the role of probiotics in this context remain less understood.

**Methods:**

C57BL/6J mice (8-week-old) were subcutaneously administered with 8 weeks of D-galactose (D-gal) or phosphate buffer solution (PBS) for aging and non-aging models, respectively, with or without 8 weeks of oral *Lacticaseibacillus rhamnosus* GG (LGG). Additionally, the impact of the condition media from LGG (LCM) was tested in macrophages (RAW 264.7 cells), microglia (BV-2 cells), and hippocampal cells (HT-22 cells).

**Result:**

Fecal microbiome analysis demonstrated D-gal-induced dysbiosis (reduced Firmicutes and Desulfobacterota with increased Bacteroidota and Verrucomicrobiota), which LGG partially neutralized the dysbiosis. D-gal also worsens cecal ligation and puncture (CLP) sepsis severity when compared with PBS-CLP mice, as indicated by serum creatinine (Scr) and alanine transaminase (ALT), but not mortality, neurological characteristics (SHIRPA score), and serum cytokines (TNF-α and IL-6). Additionally, D-gal-induced aging was supported by fibrosis in the liver, kidney, and lung; however, CLP sepsis did not worsen fibrosis. Interestingly, LGG attenuated all parameters (mortality, Scr, ALT, SHIRPA, and cytokines) in non-aging sepsis (PBS-CLP) while improving all these parameters, except for mortality and serum IL-6, in aging sepsis (D-gal CLP). For the in vitro test using lipopolysaccharide (LPS) stimulation, LCM attenuated inflammation in some parameters on RAW264.7 cells but not BV-2 and HT-22 cells, implying a direct anti-inflammatory effect of LGG on macrophages, but not in cells from the brain.

**Conclusion:**

D-gal induced fecal dysbiosis and worsened sepsis severity as determined by Scr and ALT, and LGG could alleviate most of the selected parameters of sepsis, including SAE. However, the impact of LGG on SAE was not a direct delivery of beneficial molecules from the gut to the brain but partly due to the attenuation of systemic inflammation through the modulation of macrophages.

## Introduction

Sepsis or septicemia is a life-threatening condition that often leads to death, especially in the elderly (an increasing population worldwide), which is the body’s extreme responses to infection resulting in multi-organ failure with high mortality [1,2]. A diffuse brain dysfunction caused by sepsis without evidence of brain infection, metabolic disturbance, or other encephalopathy causes is determined as sepsis-associated encephalopathy (SAE), which is found in approximately 70% of patients ranging from mild symptoms (confusion, inattention, focus deficits) to the severe characteristics (deep coma), considering the most frequent sepsis complication [3]. Although endothelial injury, blood-brain barrier defect, inflammation, neuron signaling interference, and cell apoptosis are the well-known pathogenesis of SAE [4] The impact of organisms in the gut (gut microbiota) on SAE is also mentioned as a part of the “gut-brain axis.” [5]. The gut microbiome is the composition of microorganisms in the lumen of the intestine that indicate personal health, and changing gut microbiome composition by several factors, such as diet, metabolism, and immune response, might affect the healthiness of the host, referred to as “gut dysbiosis.” [6,7]. In sepsis, gut dysbiosis is common, partly due to the intestinal epithelial damage from several factors (immune responses, hypoxemia), leading to the translocation of microbial molecules from the gut into the blood circulation (leaky gut) [8]. Not only could intestinal damage induce dysbiosis, but dysbiosis also weakens intestinal integrity and might cause sepsis [9–11]. As such, leaky gut-induced lipopolysaccharide (LPS) or endotoxin (a primary cell wall component of Gram-negative bacteria) in serum might worsen SAE [12–14] and the benefits of probiotics on sepsis are mentioned [15].

However, studies of sepsis in the elderly, especially with SAE and probiotics, are still few. Indeed, using D-galactose (D-gal) administration in mice to induce aging through increased oxidative stress is a standard aging mouse model [16–18]. Many experiments have shown that chronic administration of D-gal leads to a significant increase in reactive oxygen species (ROS) and a decrease in the activity of antioxidant enzymes, superoxide anions, and other oxidation products in various organs. Redundancy of the ROS can cause injury to numerous cells and tissues, leading to accelerated senescence in mice, which resembles natural aging symptoms in humans [19,20]. Although the impacts of *Lacticaseibacillus rhanmosus* Gorbach-Goldin (LGG), the commercially available probiotics, in aging mice using D-galactose induction are mentioned [21], the effect of aging on sepsis severity and SAE are not mentioned. Interestingly, LGG is one of the well-known beneficial probiotics [22], partly due to its ability to form biofilms in the intestines, reduce pathogenic microbes’ intestinal adherence, and maintain a pH balance unsuitable for pathogenic organisms [23]. In sepsis, LGG is also one of the common probiotics tested in rodent models [24–26] and humans [27]. However, LGG has never been tested in the sepsis of aging model, despite the higher incidence of sepsis in the elderly compared with other age groups [28]. Because LGG demonstrated an impact on both aging [21] and sepsis [29], we hypothesized that LGG might be beneficial in sepsis in the elderly and possibly directly produce some beneficial molecules to some cells in the brain. Hence, we tested the impact of LGG in aging-induced dysbiosis and on D-gal-administered mice with cecal ligation and puncture (CLP) surgery (sepsis induction) and tested LGG in the cell line of macrophages, microglia (macrophages in the brain), and hippocampal cells.

## Materials and methods

### Animal and animal model

The animal study protocol (0108/2567) following the US National Institutes of Health (NIH) animal study protocol, the Institutional Animal Care and Use Committee of the Faculty of Medicine, Chulalongkorn University approved the animal study protocol. Wildtype C57BL/6J (WT) mice were purchased from Nomura Siam (Pathumwan, Bangkok, Thailand), and 8-week-old male mice were used. The mice were housed in standard clear plastic cages (3–5 mice per cage), had free access to water and food (SmartHeart Rodent; Perfect Companion Pet care, Bangkok, Thailand), and were subject to light/dark cycles of 12/12 h in 25°C, with 50±10% relative humidity using thick paper stripes for environmental enrichment. For aging induction, daily subcutaneous injection of D-galactose (D-gal) (Sigma-Aldrich, St. Louis, MA, USA) at 120 mg/kg/mice diluted in 0.1 mL normal saline (NSS) (or NSS alone for the non-aging group) was administered for 8 weeks. In the group receiving both D-gal and probiotics, daily oral administration (gavage) was performed with 1x10^7^ CFU/mL of *Lacticaseibacillus rhamnosus* GG (LGG) in 0.3 mL NSS (or NSS alone for the control group) for 7 days before collecting feces for microbiome analysis or performing cecal ligation and puncture (CLP) for sepsis induction. Notably, subcutaneous D-gal injection and oral gavage were performed daily at 8:00 AM, and CLP was conducted at 10 AM (2 h after the last dose of D-gal and probiotics). For the surgical procedure, CLP was performed as previously described [30]. Using isoflurane anesthesia with the ligated cecum at 1 cm from the tip before puncturing with a 21-gauge needle. Mice were sacrificed at 24 h under isoflurane anesthesia (or at 96 h for survival analysis) and collected blood via cardiac puncture (spin down to separate serum and storing at -80°C until used). The internal organs were collected and stored in 10% formalin for histology evaluation. For the neurological screening, SHIRPA (The SmithKline, Harwell, Imperial College, Royal Hospital, Phenotype Assessment) score, based on abnormal behavior (score 0–1), aggression (score 0–1), contact righting reflex (score 0–1), tremor (score 0–2), body tone (score 0–2), corneal reflex (score 0–2), negative geotaxis (score 0–4), pinna reflex (score 0–2), righting reflex (score 0–3), spontaneous activity (score 0–5), toe pinch (score 0–5), visual placing (score 0–5), Mir maneuver (score 0–5) [31,32].

### Mouse sample analysis and histology

To avoid impacts of coprophagy (a habit of consuming feces from other mice in the same cage), mice from different cages were used for the fecal collection and performed microbiome analysis as previously described [33]. Briefly, 0.3 g of fecal sample per mouse was used and the feces from 3 mice were combined into a sample for the metagenomic DNA extractions using DNeasy Kit (Qiagen GmbH, Hilden, Germany) and DNA quality was assessed by nanodrop spectrophotometry using the universal prokaryotic primers 515F (5’-GTGCCAGCMGCCGCGGTAA-3’) and 806R (5’-GGACTACHVGGGTWTCTAAT-3’). The appended 5’ Illumina adapter and 3’ Golay barcode sequences were used for 16S rRNA gene V4 library construction. Then, the samples were processed using the Mothur method. For serum creatinine and alanine transaminase (ALT), QuantiChrom creatinine assays (DICR-500) and EnzyChrom alanine transaminase assay (EALT-100) (Bioassay, Hayward, CA, USA), respectively, were used. The accession number for the microbiome data is PRJNA966754. Meanwhile, serum cytokines were measured by an enzyme-linked immunosorbent assay (ELISA) (Invitrogen, Carlsbad, CA, USA). Additionally, the internal organs of mice, including livers, lungs, and kidneys, were fixed in 10% neutral buffered formalin fixation, embedded with paraffin, and cut to a 4 μm-thickness slide before staining with hematoxylin and eosin (H&E) color and evaluating with several injury scores as previously described [34]. For the liver, the injury score is based on cell congestion, cellular degeneration, cytoplasmic vacuolization, leukocyte infiltration, and cellular necrosis in 10 randomly selected fields at 200× magnification for each animal, with the following score of the damaged area per the examined field: 0 (areas less than 10%); 1 (10–25%); 2 damage (25–50%); 3 (50–75%), and 4 (75–100%), were used. Meanwhile, a subjective scale of 0 to 4 based on the inflammatory cell infiltration per high power field of the lung histopathology (HPF; 400× magnification) using 10 HPFs per mouse, including 0: no inflammatory cells,1: few cells, 2: the inflammatory cells in 1 cell layer deep; 3: the inflammatory cells in 2–4 cells deep, and 4: the inflammatory cells of > 4 cells deep, was used for lung injury. For the kidney, a score determined from the area of injury based on tubular epithelial swelling, loss of brush border, vacuolar degeneration, necrotic tubules, cast formation, and desquamation with the following scale: 0 (area of damage < 5%); 1, (5–10%); 2, (10–25%); 3, (25–50%); and 4, (> 50%), were used. For aging-induced organ fibrosis, the blue color in a Masson Trichrome Stain (MSB) stained slide using the computerized image analysis software ImageJ (Bethesda, MD, USA) in a 200x magnification field with 10 fields per sample was used, according to a previous publication [35].

### In vitro experiments

The LGG (Mead-Johnson, Evansville, IN, USA) was stored at −80°C in Man Rogosa Sharpe (MRS) broth (Oxoid, Hampshire, UK) containing 10% (vol/vol) glycerol and LGG-conditioned media (LCM) were prepared as previously described [36]. Briefly, LGG was adjusted into the concentration of 1x10^7^ CFU/mL by OD600 of 0.1 in MRS broth and incubated anaerobically for 8 h. Cell-free supernatants were filtered by a 0.22 μm membrane filter (Minisart, Sartorius Stedim Biotech GmbH, Goettingen, Germany) and concentrated by speed vacuum drying for 18–24 h. (Savant SPD1010, Thermo Scientific, United States). Cell-free concentrated pellets were resuspended in Dulbecco’s Modified Eagle’s Medium (DMEM) 500 μl and stored at -20°C until further used. Then, RAW 264.7 (macrophages), BV-2 (microglia), and HT-22 (hippocampal cells) obtained from American Type Culture Collection (Manassas, VA, USA) were cultured in Dulbecco’s Modified Eagle’s Medium (DMEM) with 1% Penicillin-Streptomycin, 10% heat-inactivated fetal bovine serum, 1% HEPES and 1% sodium pyruvate under 37°C 5% CO_2_ atmosphere for 24 h. After that, the adherent cells were harvested with 0.25% (v/v) trypsin in 1 mM EDTA (Gibco-Invitrogen, USA) and resuspended in complete media for further examination. The MTS assay (cell viability test) was performed to investigate cytotoxicity. Then, the cells were treated with the following conditions: lipopolysaccharides (LPS) 100 ng/mL, D-gal 100 mM, LPS with D-gal, LPS with D-gal with LCM, and DMEM alone as a control. Notably, the concentrations of LPS and D-gal used followed previous publications [30,37]. After being treated for 24 h, cell-free supernatants were investigated for cytokines (TNF-α IL-6 and IL-10) by ELISA assay (Invitrogen, SanDiego, CA, USA). Additionally, several genes were expressed by quantitative real-time PCR, following a previous protocol [38]. The primers were listed in **Table 1.** with the following details; *NF-κB RelA* (mouse: NM_009045), *TLR-4* (mouse: NM_021297.3), Arginase-1 (*Arg 1)* (mouse: NM_007482), *iNOS* (mouse: NM_010927), TNF-α (mouse: BC137720), *TGF-β1* (mouse: NM_011577.2), *IL-1β* (mouse: NM_008361.4), *IL-10* (mouse: NM_010548.2), and *β-actin* (mouse: NM_007393.5). The RNA extraction was performed by an RNA isolation kit (FAVORGEN Biotech Corporation, FavorPrepTM, Taiwan), and the concentration was by NanoDrop spectrophotometer (NanoDrop 1000, Thermo Fisher Scientific, Waltham, MA USA). Then, RNA was converted into cDNA by a Reverse transcription System, and qPCR was performed using the SYBR Green system (Applied Biosystem, Foster City, CA, USA). Gene expression was analyzed by 2XPowerUpTMSYBRTMGreen Master Mix (Thermo Fisher Scientific, Waltham, MA, USA) according to the manufacturer’s instructions with QuantStudio® 6 Real-Time PCR system (Applied Biosystems™, Life Technology Corporation, CA, USA).

**Table 1:** List of primers used in this study.

Primers	Forward	Reverse
Nuclear factor-κB (NF-κB RelA)	5’-CTTCCTCAGCCATGGTACCTCT-3’	5’ -CAAGTCTTCATCAGCATCAAACTG-3’
Toll like receptor 4 (TLR-4)	5’-GGCAGCAGGTGGAATTGTAT-3’	5’ -AGGCCCCAGAGTTTTGTTCT-3’
Arginase-1 (Arg-1)	5’ -CTTGGCTTGCTTCGGAACTC-3’	5’-GGAGAAGGCGTTTGCTTAGTT-3’
Inducible nitric oxide synthase (iNOS)	5’-ACCCACATCTGGCAGAATGAG-3’	5’-AGCCATGACCTTTCGCATTAG-3’
Transforming Growth Factor-β (TGF-β)	5’ -CAGAGCTGCGCTTGCAGAG-3’	5’ -GTCAGCAGCCGGTTACCAAG-3’
Interleukin-1β (IL-1β)	5’-GAAATGCCACCTTTTGACAGTG-3’	5’ -TGGATGCTCTCATCAGGACAG-3’
Interleukin-10 (IL-10)	5’-GCTCTTACTGACTGGCATGAG-3’	5’ -CGCAGCTCTAGGAGCATGTG-3’
Resistin-like molecule-α (FIZZ-1)	5’ -GCCAGGTCCTGGAACCTTTC-3’	5’ -GGAGCAGGGAGATGCAGATGA-3’
Tumor necrosis factor α (TNF-α)	5’-CCTCACACTCAGATCATCTTCTC-3’	5’ -AGATCCATGCCGTTGGCCAG-3’
Interferon gamma (IFN-**γ**)	5’-ACTGACTTGAATGTCCAACGCA-3’	5’-ATCTGACTCCTTTTTCGCTTCC-3’
β-actin	5’-CGGTTCCGATGCCCTGAGGCTCTT-3’	5’-CGTCACACTTCATGATGGAATTGA-3’

### Statistical analysis

All data was analyzed by Graph Pad Prism version 10.0 software (La Jolla, CA, USA) as means ± standard errors (SE). The difference between groups was analyzed using the Student’s T-test and one-way analysis of variance (ANOVA) with Tukey’s test to examine two groups or multiple groups, respectively, with the P value of 0.05 as a statistical significance.

## Result

### Probiotics attenuated aging-induced fecal dysbiosis

**Fig 1:**
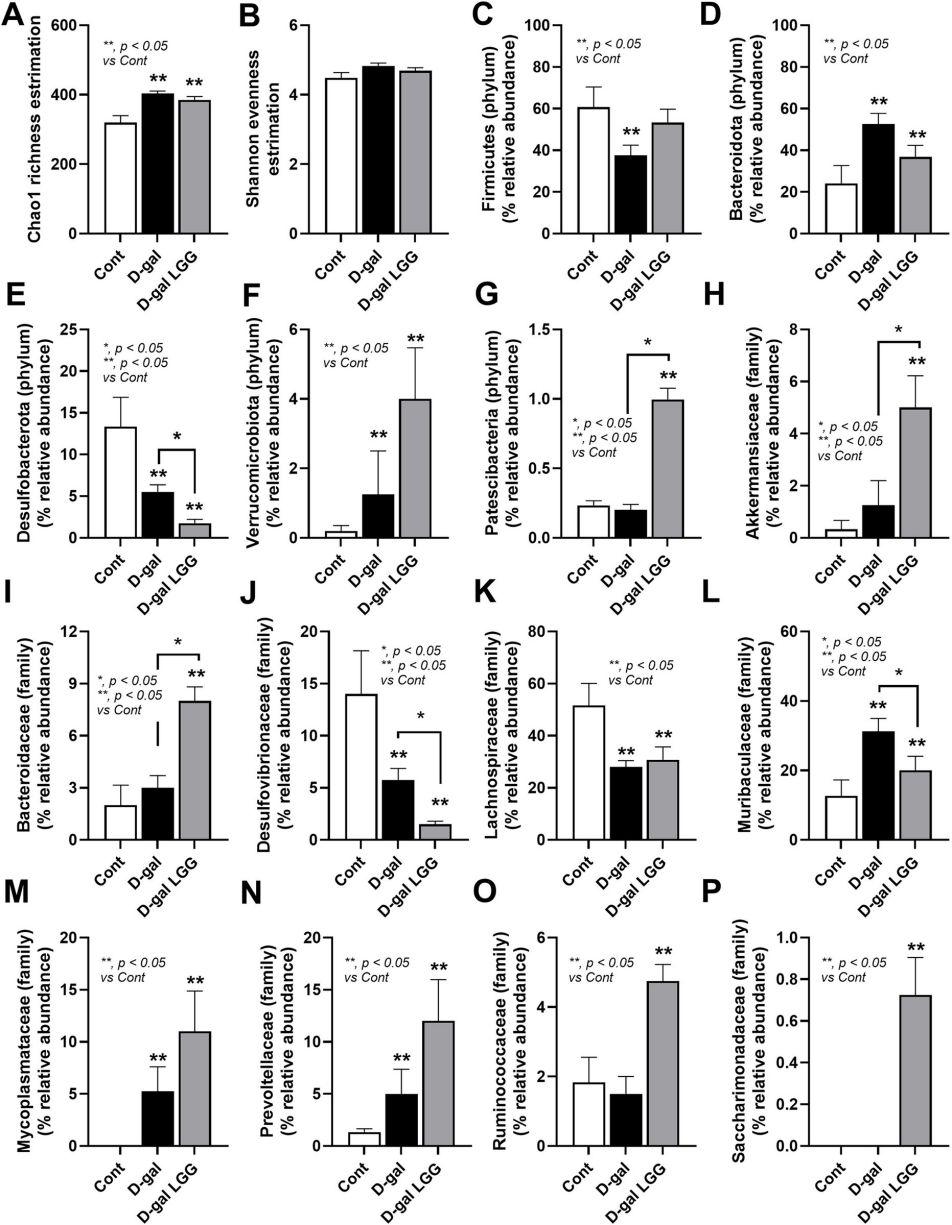
Fecal microbiome analysis from D-galactose (D-gal)-administered mice with or without probiotics (LGG) and control mice as indicated by alpha diversity (Chao-1 and Shannon) (A, B), bacterial abundance in phylum level, including Firmicutes (also known as Bacillota) (mostly beneficial Gram-positive anaerobes for humans), Bacteroidota (mostly Gram-negative anaerobes with beneficial and harmful properties to humans), Desulfobacteroidota (thermophilic sulfate-reducing bacteria with possible pathogenic property to humans), Verrucomicrobiota (Gram-negative bacteria with some beneficial properties), and Patescibacteria (nanobacteria or ultra-small bacteria) (C-H), and family level, including Akkermansiaceae (beneficial bacteria in phylum Verrucomicrobiota), Bacteroidaceae (mostly Gram-negative anaerobes), Desulfovibrionaceae, Lachnospiraceae (obligately beneficial anaerobes in phylum Firmicutes), Muribacuaceae (high abundant bacteria in mammal gut from phylum Bacteroidota), Mycoplasmataceae (Mycoplasma and Ureaplasma), Prevotellaceae (from phylum Bacteroidota), Ruminococcaceae (strictly anaerobes in Firmicutes group), and Saccharimodanaceae (also known as Saccharibacteria, the group of *Candidatus Saccharimonas*, the uncultured bacteria discovered through 16S rRNA sequencing) (I-P) are demonstrated (n = 3 for control and n = 4/ group for D-gal and D-gal + LGG). *, *p < 0*.*05*; **, *p < 0*.*05* vs control.

**Fig 2:**
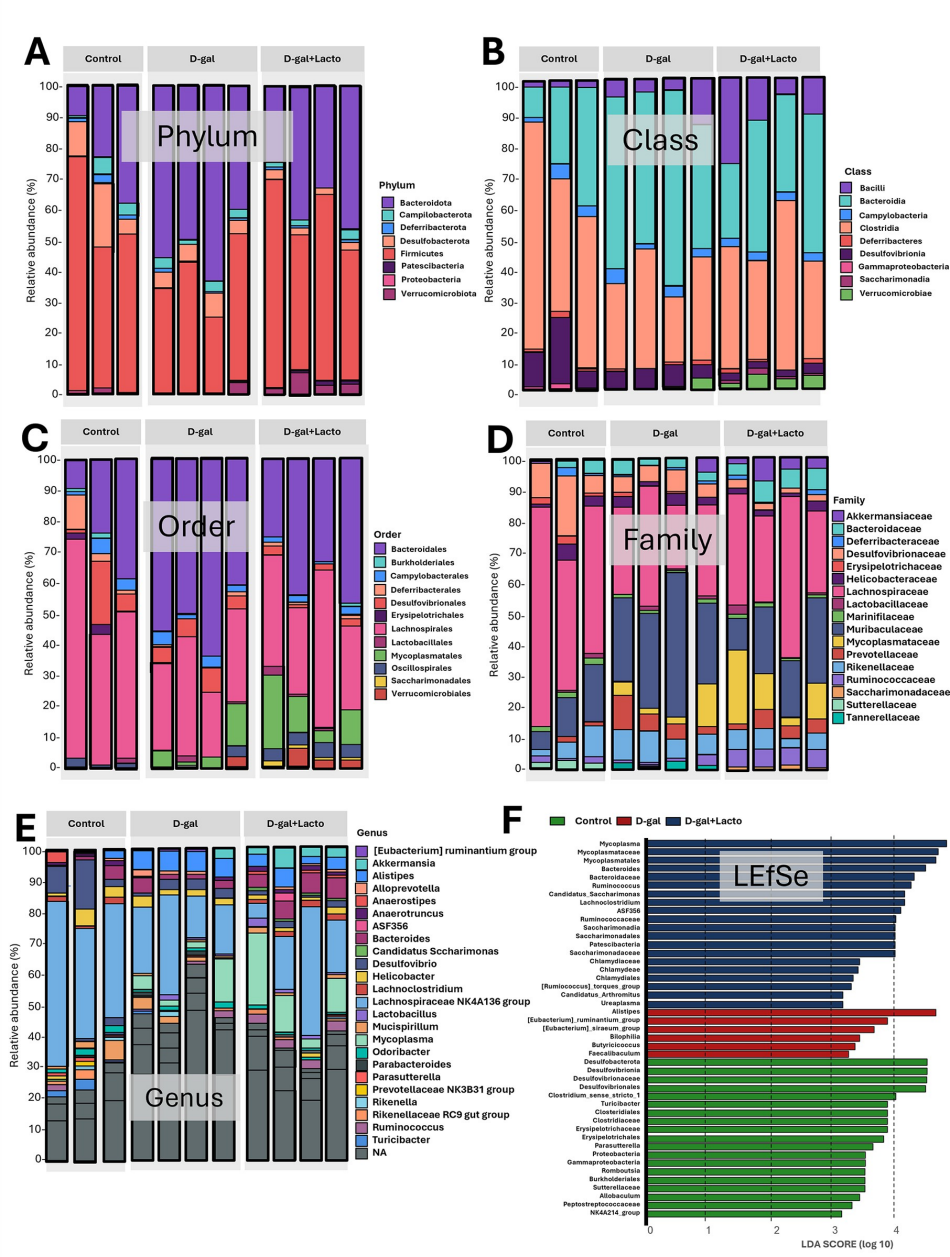
Fecal microbiome analysis from D-galactose (D-gal)-administered mice with or without probiotics (LGG) and control mice as indicated by bacterial abundance in the phylum, family, class, order, family, and genus level (A-E) with the Linear discriminant analysis Effect Size) (LEfSe; the representative bacteria for each group) are demonstrated (n = 3 for control and n = 4/ group for D-gal and D-gal + LGG). Notably, LEfSe is one of the parameters demonstrating differences among groups because specific bacteria are usually better able to grow in the specific microenvironment.

**Fig 3:**
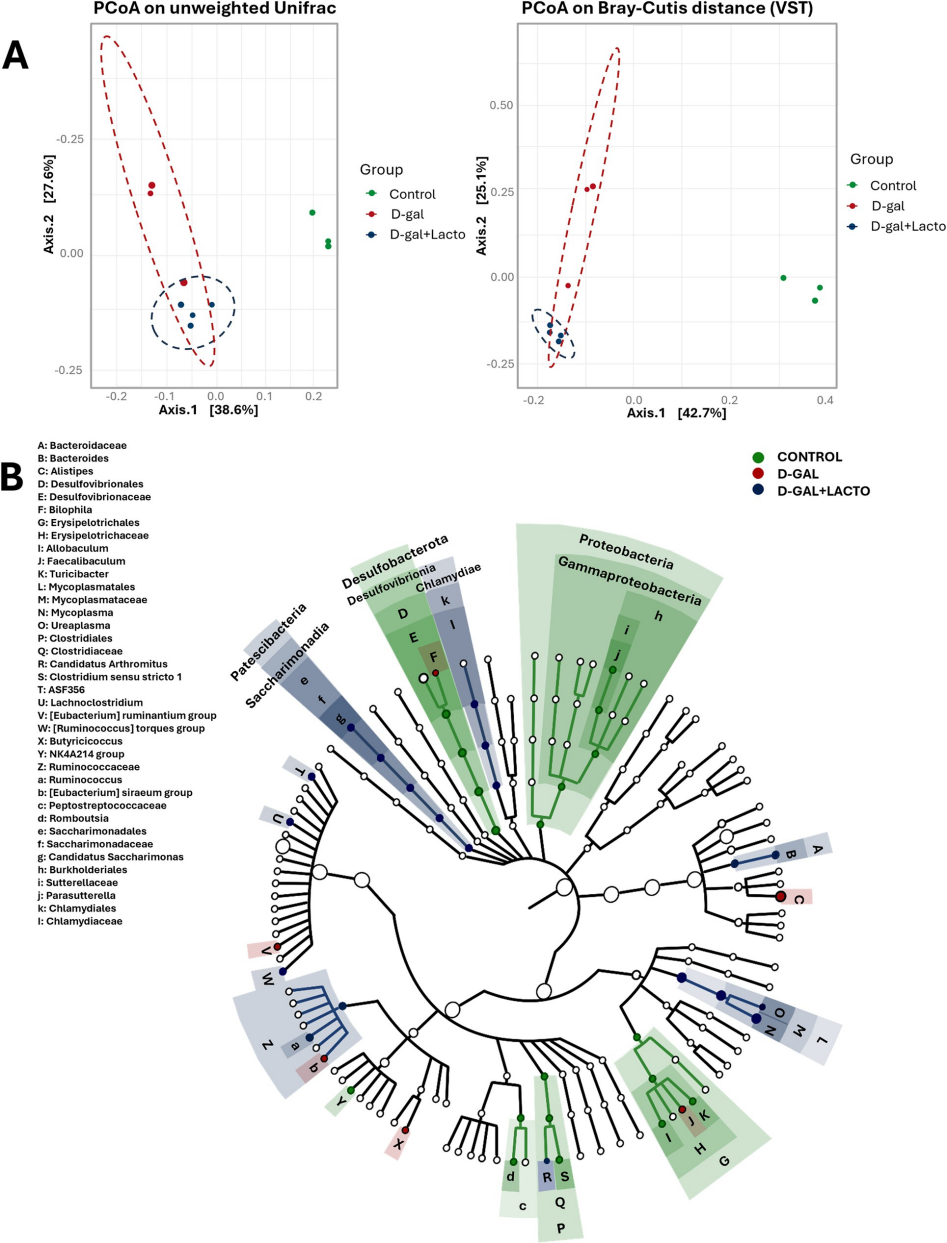
Fecal microbiome analysis from D-galactose (D-gal)-administered mice with or without probiotics (LGG) and control mice as indicated by principal coordinates analysis (PCoA; the differences among groups as represented by distances from the axis) using unweight UniFrac (considering the presence and absence of species) (A), Bray-Curtis distance (considering abundance information) (B), and Cladogram (a diagram showing relationships between species) (C) are demonstrated. Notably, a PCoA plot is used to easily demonstrate differences among groups, which might consist of several aspects into only 2 parameters, including the distance from the x and y axes.

Despite the previously mentioned impacts of *Lacticaseibacillus rhamnosus* (LGG) (the commercially available probiotics) in aging-induced fecal dysbiosis from D-galactose (D-gal)-administered mice [39], we tested the benefits of LGG before using it in sepsis. As such, D-galactose increased Chao-1 richness estimation (the number of observed species in the population) without the changes in Shannon evenness estimation (the abundance of each species relative to other species within the community), and LGG could not neutralize these values (**Fig 1A and 1B**). Compared with the control, fecal dysbiosis in D-gal-induced aging was demonstrated by an increase in bacteria in the phylum Firmicutes (mostly beneficial Gram-positive anaerobes) and Verrucomicrobiota (Gram-negative bacteria, including potential probiotics, especially *Akkermansia muciniphila*) [40]. with an elevation in Bacteroidota (mostly Gram-negative anaerobes) and Desulfobacteroidota (intracellular thermophilic sulfate-reducing bacteria with potential pathogenicity) [41]. (**Fig 1C–1F**). With LGG, only Firmicutes could be neutralized, while LGG with D-gal elevated Patescibacteria, referred to as “candidate phyla radiation” (a primarily uncultivated bacteria, described as nanobacteria or ultra-small bacteria) [42]. (**Fig 1C and 1G**). The families of bacteria from these phyla with a significant level among groups were demonstrated in graph presentation (**Fig 1H–1P**), and bacterial abundance from microbiota analysis was demonstrated in **Fig 2A–2E**. Additionally, the representative bacteria of the groups, as determined by LEfSe (Linear discriminant analysis Effect Size), showed different bacteria in each group (**Fig 2F**). For example, *Desulfobacteria* represented the control group, while *Alistepes* spp. and Mycoplasma represented D-gal and D-gal with LGG mice (**Fig 2F**). Moreover, the beta diversity as determined by the principal coordinates’ analysis (PCoA; the demonstration of differences among groups through distances from the axis) using Unweighted UniFrac (the distance from axis based on the presence and absence of species) and Bray-Curtis (the distance from axis based on abundance of species) and cladogram indicated the differences among groups (**Fig 3A and 3B**). These data supported fecal dysbiosis in D-gal-induced aging mice and the impacts of LGG in dysbiosis attenuation.

### D-galactose-induced aging worsened sepsis and reduced the effectiveness of probiotics

**Fig 4:**
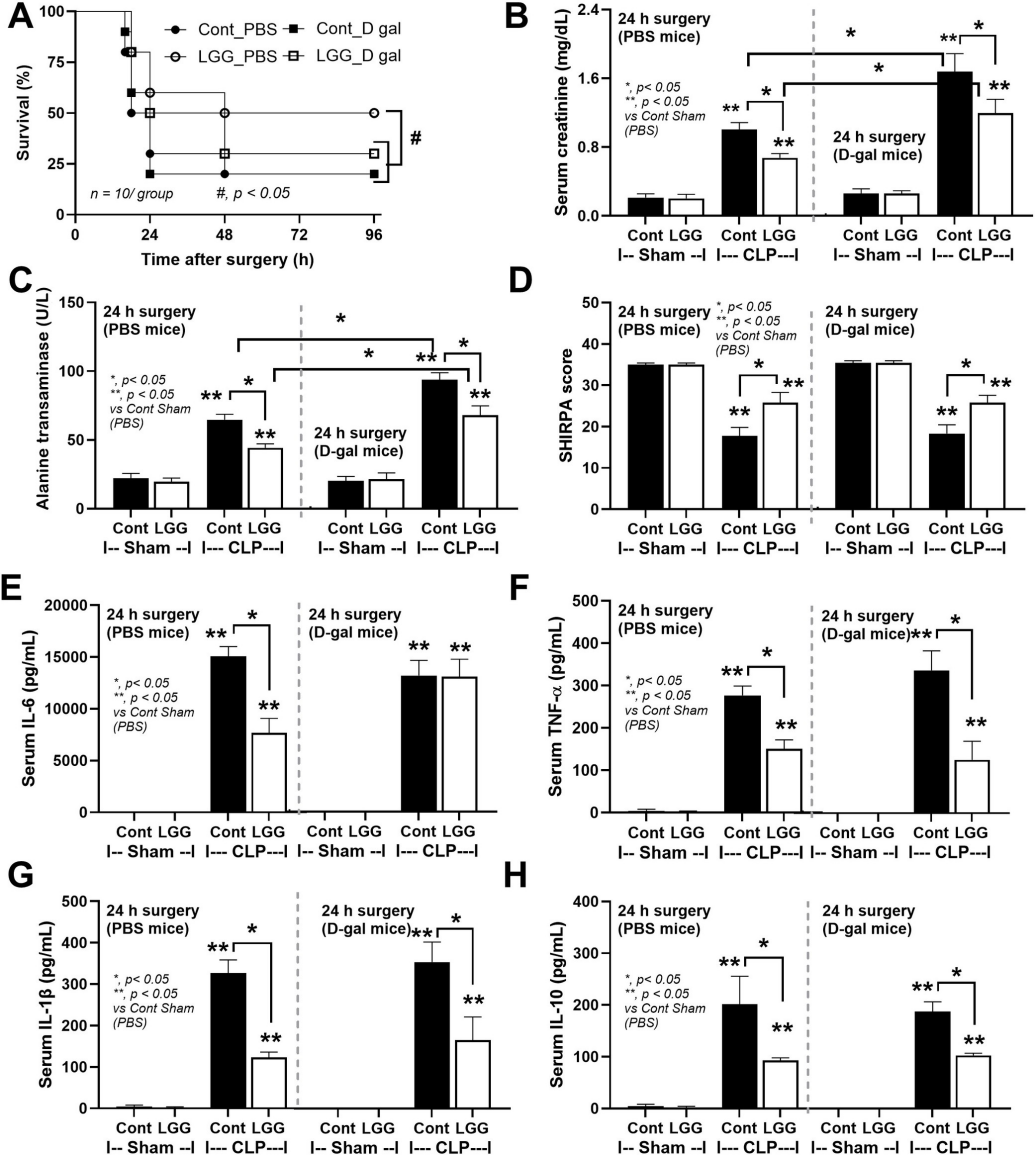
Characteristics of mice with cecal ligation and puncture (CLP) sepsis induction or sham surgery in aging model using D-galactose (D-gal)-administered mice with or without probiotics (LGG) versus non-aging model using phosphate buffer solution (PBS)-administered mice with or without probiotics as indicated by survival analysis (A), serum creatinine for kidney function (B), alanine transaminase (ALT) for liver function (C), SHIRPA score for neurological observation (D), and serum cytokines (IL-6 and TNF-a) (E, F) are demonstrated (n = 10/group for survival study and n = 5-7/group for other parameters). *, *p < 0*.*05*; **, *p < 0*.*05* vs control sham PBS. Notably, the SHIRPA (The SmithKline, Harwell, Imperial College, Royal Hospital, Phenotype Assessment) score is used to determine differences of the mouse phenotypes with a total of 37 scores with the neurological aspects during the observation (see method).

**Fig 5:**
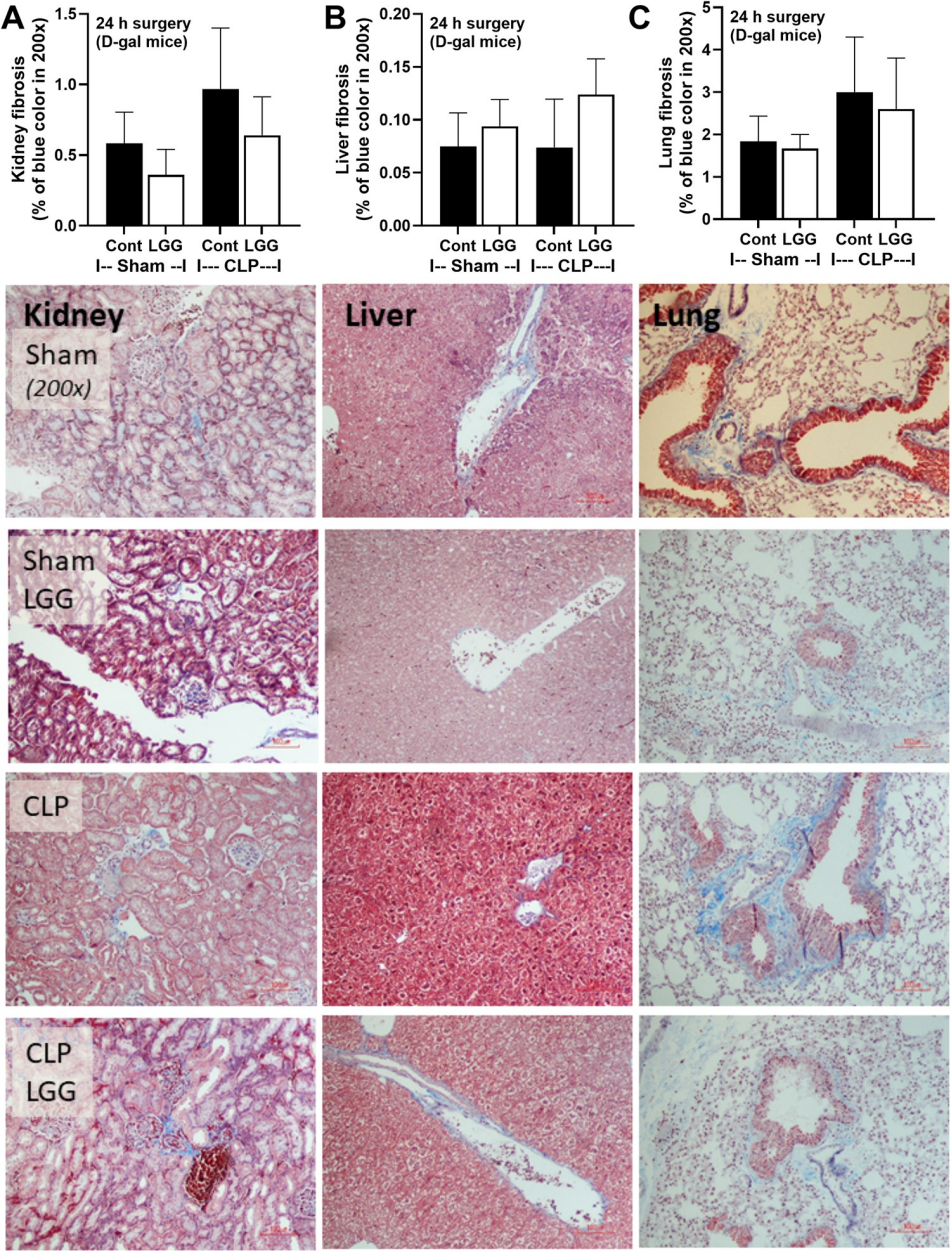
Characteristics of fibrosis in liver, kidney, and lung from D-galactose (D-gal)-administered mice with or without probiotics (LGG) with cecal ligation and puncture (CLP) sepsis induction or sham surgery as indicated by histological area (A-C) and the representative Masson’s trichrome-stained pictures are demonstrated (n = 5-7/group).

**Fig 6:**
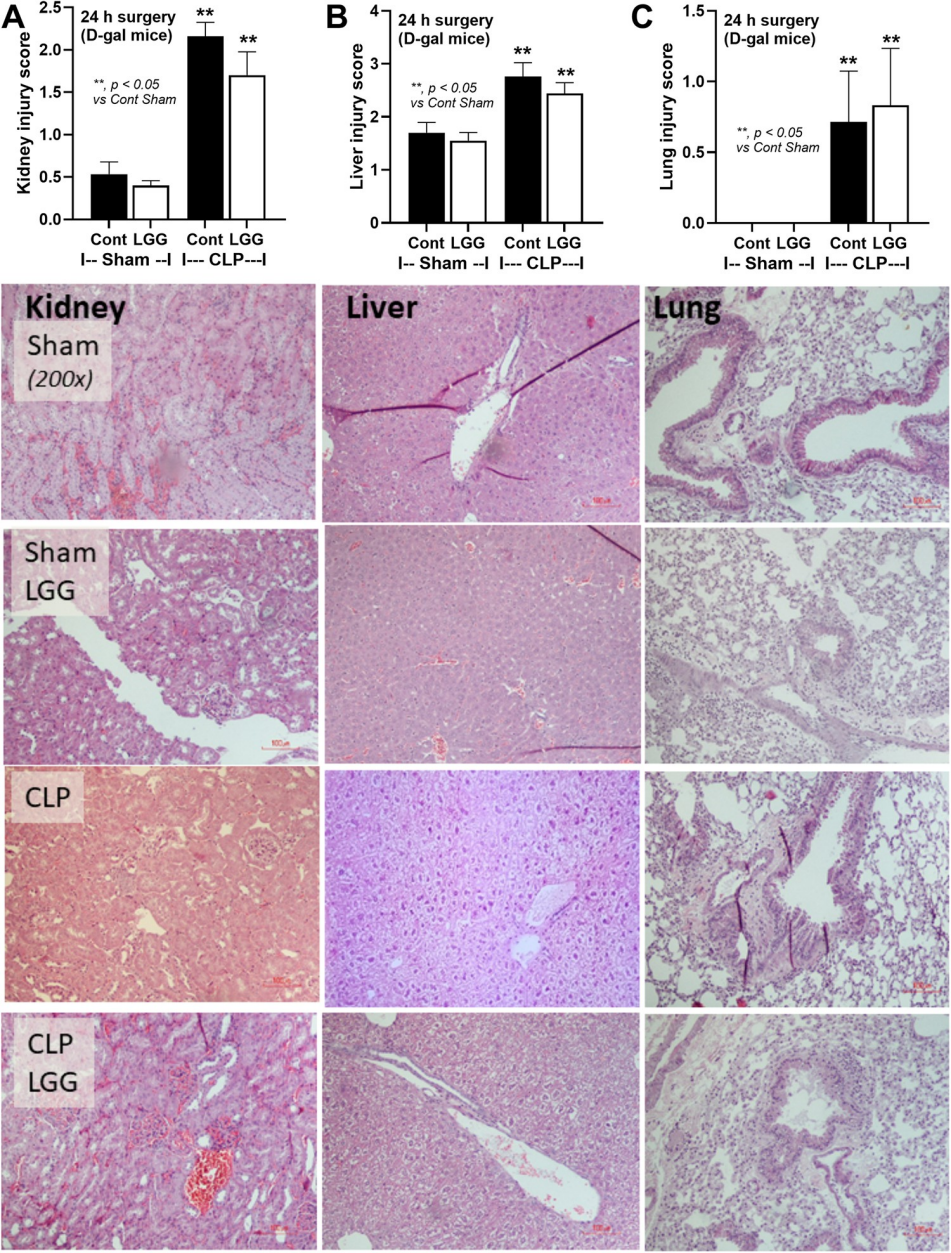
Characteristics of histological injury in liver, kidney, and lung from D-galactose (D-gal)-administered mice with or without probiotics (LGG) with cecal ligation and puncture (CLP) sepsis induction or sham surgery as indicated by histological score (A-C) and the representative Hematoxylin and eosin-stained pictures are demonstrated (n = 5-7/group). **, *p < 0*.*05* vs control sham PBS.

Because of i) the demonstrable effect of LGG on dysbiosis attenuation (**Figs 1–3**), and ii) the well-known impacts of dysbiosis in sepsis and benefits of probiotics in sepsis [8], LGG was further used in the sepsis model. Accordingly, aging induction by D-gal worsened sepsis severity only in renal and liver injury, but not mortality, encephalopathy (SHIRPA score), and serum cytokines (IL-6 and TNF-α) (**Fig 4A–4H**). Interestingly, LGG attenuated all these sepsis parameters in PBS-administered CLP mice (non-aging sepsis) (**Fig 4A–4H**). In aging D-gal-administered mice, LGG attenuated most of these parameters, except for mortality and serum IL-6 (**Fig 4A–4H**). On the other hand, fibrosis in the liver, kidney, and spleen (Masson’s Trichrome staining) was demonstrated only in mice with 8-wk-administration of D-gal (**Fig 5A–5C**) but not in PBS-administered mice and sepsis induction did not affect the severity of fibrosis. Meanwhile, LGG could not attenuate sepsis-induced injury in these organs (**Fig 6A–6C**) despite improved renal function as determined by serum creatinine (**Fig 4B**).

### The condition media from probiotics attenuated LPS-induced inflammation in macrophage cell lines but not microglia and hippocampal cells

**Fig 7:**
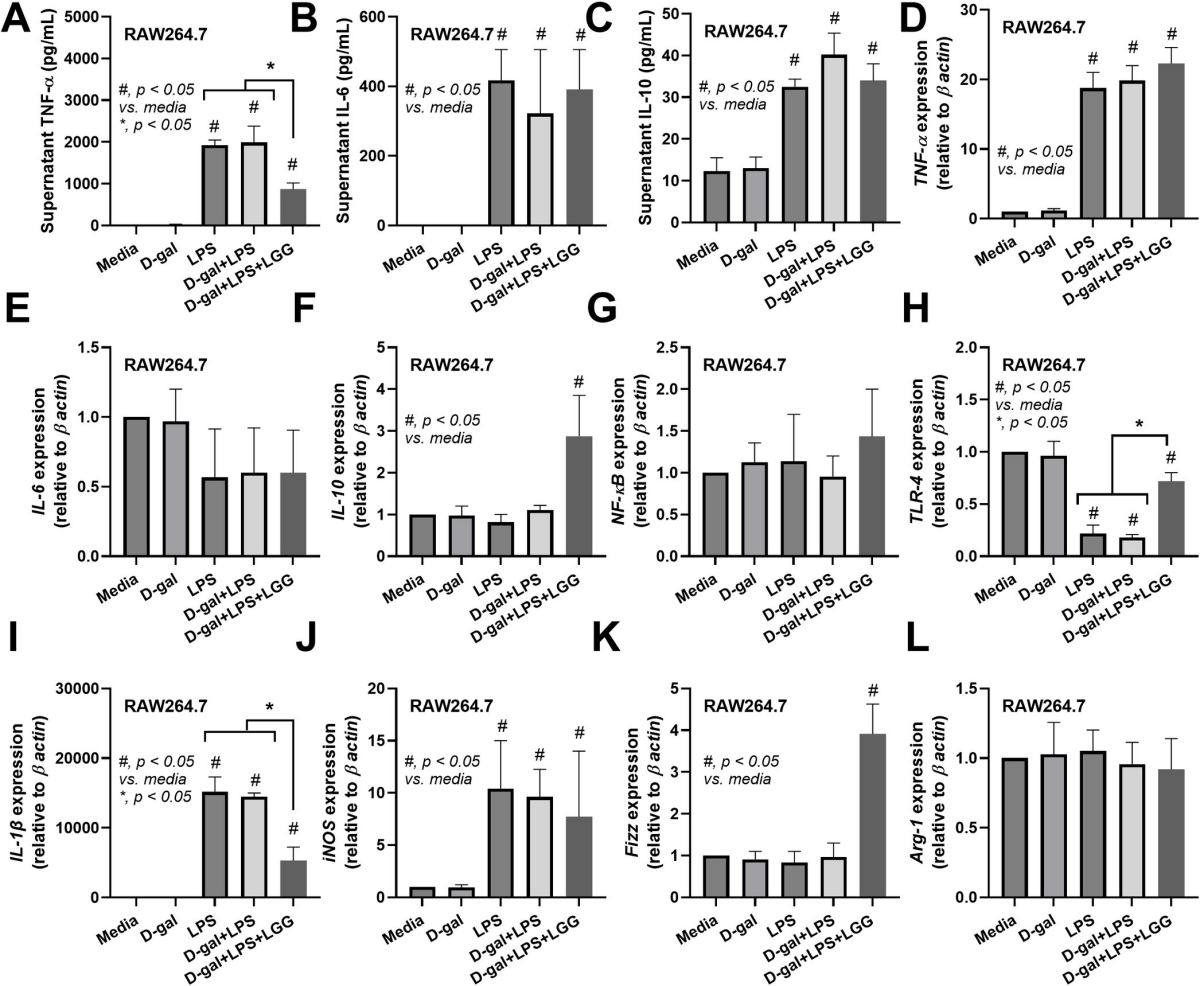
Characteristics of macrophage cell line (RAW 246.7 cells) after stimulation by D-galactose (D-gal), lipopolysaccharide (LPS), D-gal plus LPS (D-gal+LPS), D-gal+LPS with condition media of probiotics (D-gal+LPS+LGG), and control media (media) as indicated by supernatant cytokines (TNF- α IL-6, and IL-10) (A-C) and gene expression (D-F), inflammatory signaling (*NF-κB* and *TLR-4*) (G, H), M1 pro-inflammatory macrophage polarization genes (*IL-1ß* and *iNOS*) (I, J), and M2 anti-inflammatory macrophage polarization genes (*Fizz-1* and *Arg-1*) (K, L) are demonstrated (triplicated isolated experiments were performed). *, p < 0.05 vs control sham PBS, #, p< 0.05 vs media.

Due to i) the attenuation of encephalopathy (SHIRPA score) and systemic inflammation by LGG (**Fig 4D–4H**), ii) the well-known importance of macrophages on sepsis inflammation [43–45], iii) the influence of microglial cells in stimulating other cells to respond to inflammatory cytokines during sepsis [46–49], and iv) the significant and prominent impact on the hippocampus during sepsis and SAE [50,51], LGG might produce some beneficial molecules toward macrophages and cells in the brain. In macrophages (RAW264.7 cells), LPS induced inflammatory macrophages, as indicated by supernatant cytokines (TNF-α, IL-6, and IL-10), upregulation of the *TNF-α* gene, but not *IL-6*, *IL-10*, and *NF-κB*, and downregulation of *TLR-4* when compared with control with M1 pro-inflammatory macrophage polarization (elevated *IL-1β* and *iNOS*) with an unchanged in the genes of M2 anti-inflammatory macrophage polarization (*Fizz-1* and *Arg-1*) (**Fig 7A–7L**). Although the additional incubation of LPS and D-gal did not strengthen the LPS-induced pro-inflammation effect, the condition media of LGG (LCM) partially reduced the pro-inflammation effect, as indicated by reduced supernatant TNF-α, upregulated *IL-10*, neutralized an impact on *TLR-4*, downregulated *IL-1β*, and upregulated *Fizz-1* (**Fig 7A, 7F, 7H, 7I and 7K**).

**Fig 8:**
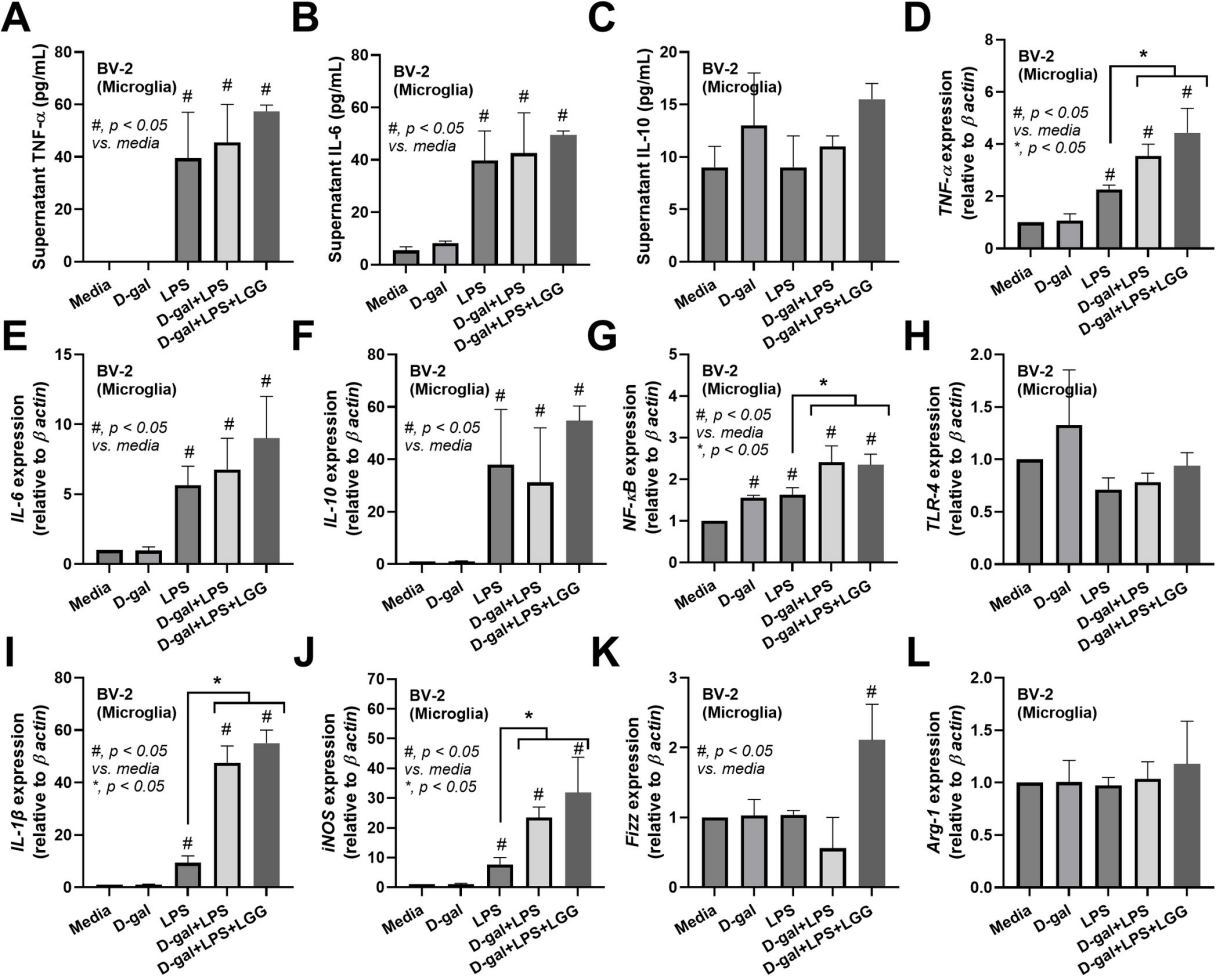
Characteristics of microglia cell line (BV-2 cells) after stimulation by D-galactose (D-gal), lipopolysaccharide (LPS), D-gal plus LPS (D-gal+LPS), D-gal+LPS with condition media of probiotics (D-gal+LPS+LGG), and control media (media) as indicated by supernatant cytokines (TNF- α IL-6, and IL-10) (A-C) and gene expression (D-F), inflammatory signaling (*NF-κB* and *TLR-4*) (G, H), M1 pro-inflammatory macrophage polarization genes (*IL-1ß* and *iNOS*) (I, J), and M2 anti-inflammatory macrophage polarization genes (*Fizz-1* and *Arg-1*) (K, L) are demonstrated. (triplicated isolated experiments were performed for all figures). *, p < 0.05 vs control sham PBS, #, p< 0.05 vs media.

**Fig 9:**
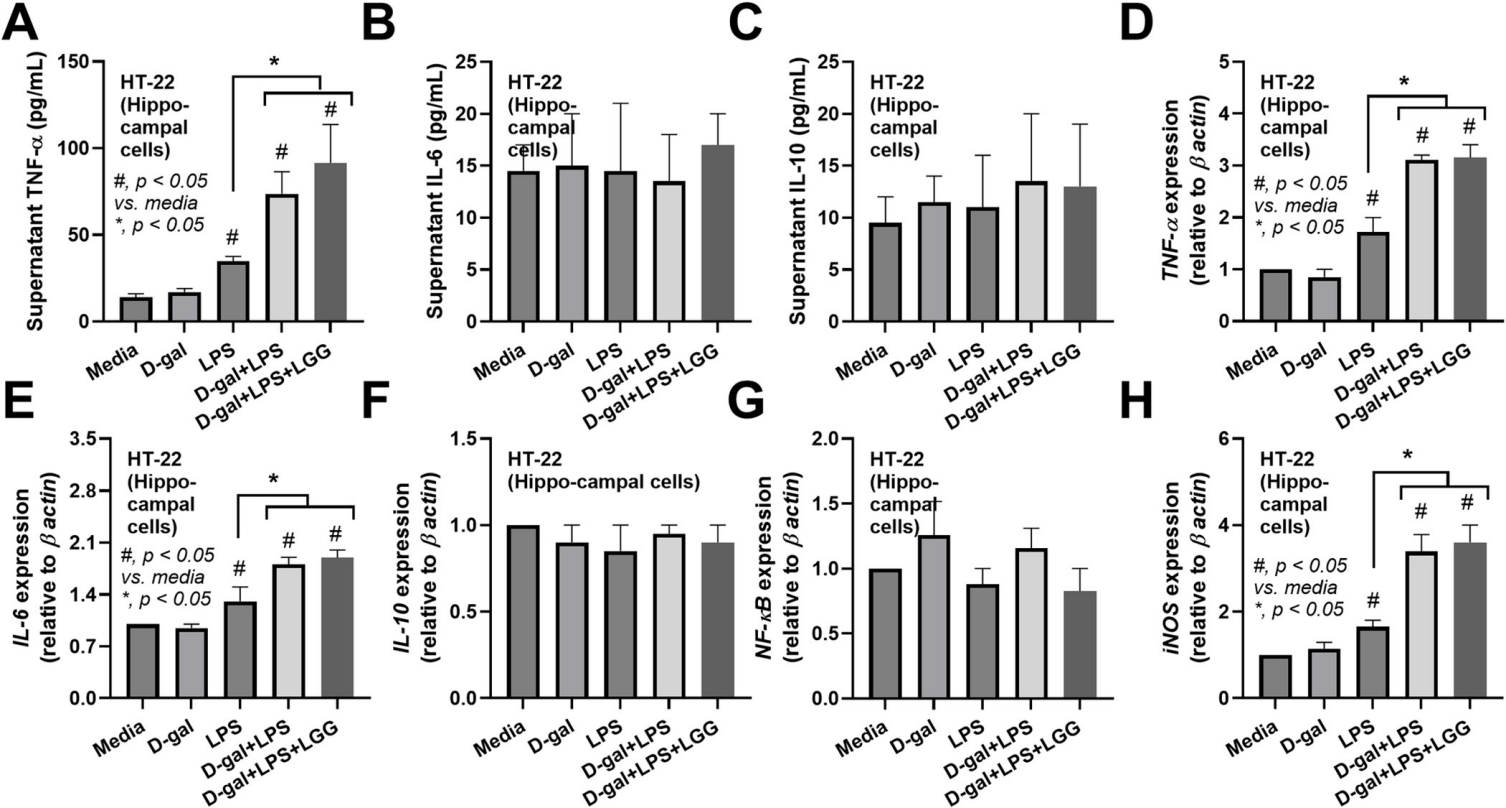
Characteristics of hippocampal cell line (HT-22 cells) after stimulation by D-galactose (D-gal), lipopolysaccharide (LPS), D-gal plus LPS (D-gal+LPS), D-gal+LPS with condition media of probiotics (D-gal+LPS+LGG), and control media (media) as indicated by supernatant cytokines (TNF- α IL-6, and IL-10) (A-C) and gene expression (D-F), *NF-κB* (G) and *iNOS* (H) are demonstrated (triplicated isolated experiments were performed for all figures). *, p < 0.05 vs control sham PBS, #, p< 0.05 vs media.

**Fig 10:**
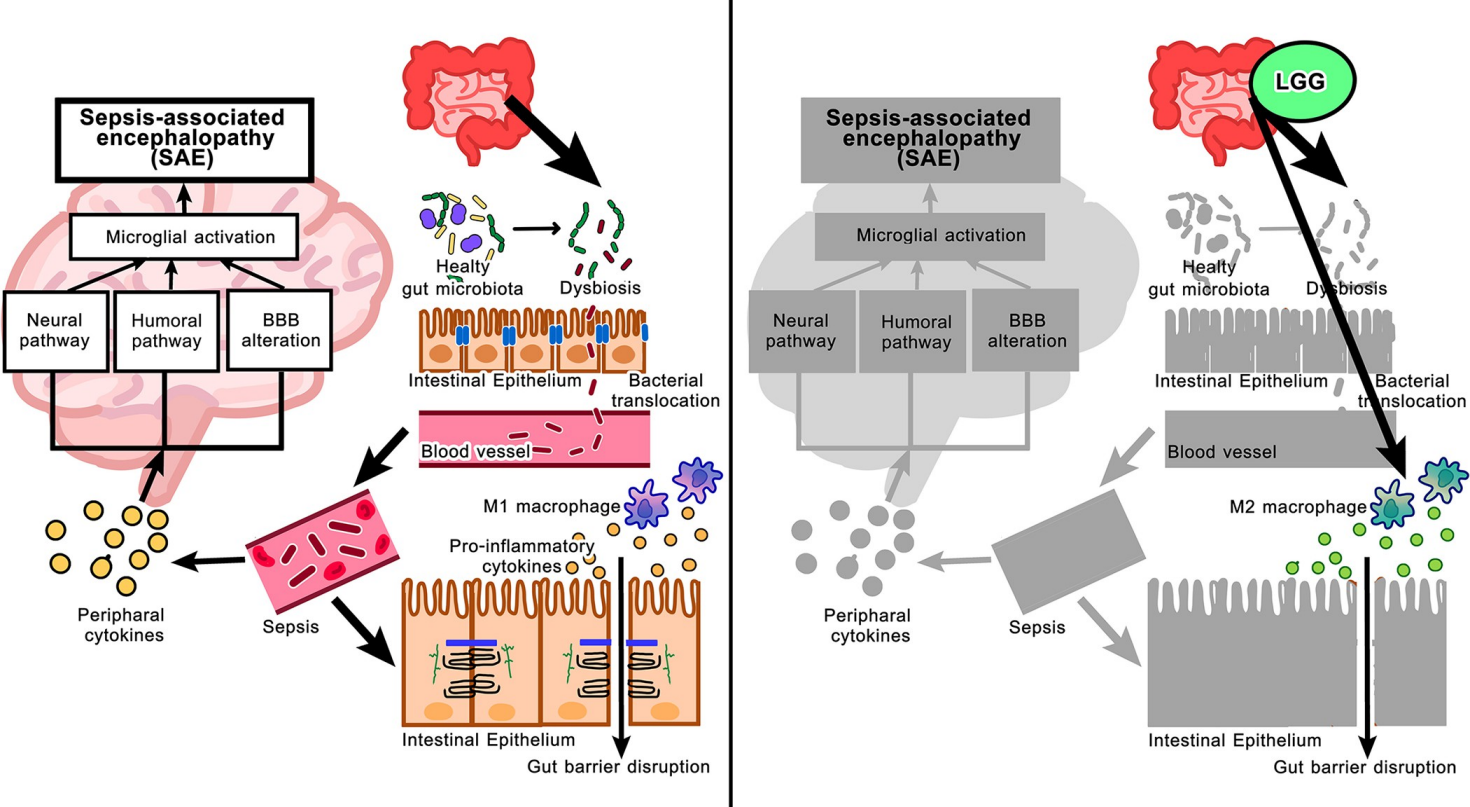
The working hypothesis demonstrates that gut dysbiosis (increased pathogens) leads to intestinal barrier dysfunction (enterocyte tight junction damage) and secretion of inflammatory cytokines of cells that possibly stimulate microglia in the brain (neural and humoral pathways with blood-brain barrier alteration), leading to sepsis-associated encephalopathy (SAE) **(left-sided image)**. With probiotics (LGG), the leaky gut attenuation and enhanced M2 macrophage polarization might reduce inflammation and indirectly improve SAE **(right-sided image)**.

Because of the well-known sepsis-induced leaky gut, which might allow the translocation of large molecular weight molecules produced from LGG in the gut into the blood circulation [52,53] LCM might contain some molecules that directly benefit the cells in the brain. Hence, LCM was tested in microglia and hippocampal cells. Although microglia are macrophages in the brain, the microglia cell line (BV-2) responses differed from those of RAW264.7 cells (**Fig 8A–8L**). As such, LPS similarly induced pro-inflammation in microglia, as indicated by supernatant cytokines (TNF-α, IL-6, but not IL-10), upregulation of *TNF-α IL-6*, *IL-10*, and *NF-κB*, and unchanged *TLR-4* when compared with control with M1 polarization (elevated *IL-1β* and *iNOS* with an unchanged in *Fizz-1* and *Arg-1*) (**Fig 8A–8L**). In microglia, D-gal demonstrated a possibly mildly additive effect on LPS, as D-gal plus LPS induced higher *IL-1β* and *iNOS* when compared with LPS alone (**Fig 8I and 8J**). The addition of LCM in D-gal plus LPS did not alter these parameters, except for an up-regulation of Fizz-1 (**Fig 8K**). In hippocampal cells (HT-22), LPS induced pro-inflammation, as indicated by the supernatant cytokine TNF-α and the upregulation of *TNF-α*, *IL-6*, and *iNOS* which could not be altered by either D-gal or LCM (**Fig 9A–9H**). Thus, the effect of LCM against LPS could be demonstrated only in macrophages. Still, neither microglia nor brain cells, and an anti-inflammatory effect of LGG in mice might indirectly improve brain function (**Fig 10**).

## Discussion

D-galactose administration induced gut dysbiosis and worsened sepsis mortality without impacting SAE; however, probiotics effectively attenuated sepsis severity, partly through anti-inflammatory macrophages, without a direct effect on brain cells (microglia and hippocampal cells).

### Dysbiosis in D-galactose-induced aging and impacts of probiotics

Aging-induced dysbiosis is a natural process, possibly due to the deterioration in the functions of immune cells, especially dendritic cells, and T cells, caused by aging, resulting in increased susceptibility to bacterial infection in the elderly [54]. Despite a multi-factorial involvement in natural aging (telomere damage, stress induction, DNA damage, and cell cycle arrest [55], aging-induced pro-oxidant is one of the important factors leading to the use of D-galactose in the rodent model [16]. Galactose is one of the common monosaccharides, similar to glucose, glyceraldehyde, fructose, ribose, and xylose, that are categorized as “reducing sugars” due to the easy contribution of electrons from the aldehyde group (act as “reducing agent”) and forming carboxylic acid (oxidation process). There are 2 enantiomers, including Dextrogalactose (D-galactose) and Levogalactose (L-galactose), and only D-gal, but not L-gal, can be metabolized through the Leloir pathway. D-galactose is also the most common form found in nature [56]. Unlike glucose, D-gal easily reacts with free amines of amino acids to form advanced glycation end products through nonenzymatic glycation, and the oversupply of D-gal elevates reactive oxygen species (ROS), causing pre-mature aging in rodents [17]. Here, D-gal-induced aging was supported by enhanced fibrosis in several organs following previous publications [16,57]. However, the alteration of several aging-associated genes [58] was not explored here, partly because the physiology of aging was not the study’s primary objective, which was focused on sepsis in aging conditions. Further exploration of aging-associated genes will be interesting. Regarding gut dysbiosis, D-gal-induced dysbiosis, perhaps through the interference of ROS in several immune cells [59], in our model was indicated by a decrease in Firmicutes and Desulfobacteria (beneficial bacteria) with an increase in Bacteroidota and Verrucomicrobiota (Gram-negative bacteria acting as a source of endotoxin) similar to a previous study describing D-gal-induced dysbiosis through the reduced *Erysipelotrichia* and *Clostridia* classes (*Clostridiales*) and *Erysipelotrichales* orders in the phylum Firmicutes [60]. The upregulation of Verrucomicrobiota is also known to influence macrophage polarization towards the anti-inflammatory M2 polarization [61] partly through the enhanced production of short-chain fatty acids and other metabolites [62,63]. With probiotics, there was a neutralization of Firmicutes (the high abundance bacteria in healthy conditions) [64] and an elevation of Patescibabacteria (ultra-small bacteria with uncertain effects) [65], indicating some effects on dysbiosis attenuation that might be worth a further test on sepsis.

### Sepsis worsened by D-galactose administration and was partially attenuated by probiotics

D-galactose administration has been widely used as an induced aging model, which could result in dysbiosis and an imbalance of gut microbiome composition by decreasing beneficial bacteria and increasing pathogenic bacteria [66]. Dysbiosis could reduce gut barrier integrity and increase gut permeability, allowing bacterial translocation into the bloodstream, triggering systemic inflammation, and contributing to sepsis [67,68]. Despite a well-known enhanced sepsis severity in the elderly [69], elevated sepsis severity in our D-gal mice was only serum creatinine and liver enzyme, but not mortality, neurological score, and serum cytokines, perhaps due to the limitation of pro-oxidant-induced aging compared with natural aging. Indeed, aging-induced organ fibrosis was demonstrated in D-gal mice’s liver, kidney, and lung. In 16-week-old control mice, sepsis induction could not induce fibrosis, partly because of the lack of ROS to initiate fibrosis and duration of the inflammation from sepsis is not long enough to induce fibrosis [70]. The fibrosis in several organs in D-gal mice might be, at least in part, responsible for the more severe sepsis in mice with aging induction. Among several probiotics, *Lacticaseibacillus rhanmosus* GG (LGG)’s ability to reduce epithelial damage and decrease apoptosis [71], partly due to several molecules in the supernatant, is mentioned [72,73] together with interference in macrophages [74]. Impacts of sepsis-induced dysbiosis and leaky gut caused by elevated pathogenic organisms [75,76] and damage of intestinal tight junctions [77,78] are mentioned, similar to previous publications on LGG-induced anti-inflammatory effect in sepsis [26]. Here, LGG effectively attenuated sepsis as evaluated by all selected parameters in non-aging PBS-administered mice, while LGG neither reduced serum IL-6 nor improved organ injury in aged mice. This could be due to the altered immune responses in the elderly, where chronic inflammation and immune senescence dampen the efficacy of probiotics in the younger mice. Although SHIRPA score might not be sensitive enough to detect the severity of sepsis-associated encephalopathy (SAE) between aging and non-aging mice, LGG improved SHIRPA score in sepsis mice from both groups. According to the well-known impact of inflammatory cytokines in SAE, including neural pathway (cytokine impact on triggering cholinergic anti-inflammatory reflex, which inhibited cytokine production by releasing acetylcholine) [79], humoral pathway (cytokines entered the circumventricular organs (CVOs) that stimulated the autonomic nervous system, neuroendocrine system, and somatic nervous system) [80], and blood-brain barrier alterations (stimulated cerebral endothelial cells release TNF-α, IL-1β, and TGF-β which disrupted the barrier permeability) [81], the interference of LGG on cytokine production in several cells might be necessary. The LGG-induced M2 macrophages with the upregulated *IL-10*, but not at the protein level, support the role of mRNA translation [82], which might be earlier than the synthesis of the protein level [82].

### Probiotics attenuated SAE through anti-inflammatory macrophages but did not have a direct impact on cells in the brain

Because sepsis-induced leaky gut could allow the translocation of large microbial molecules, especially LPS (approximately 65 kDa), from the gut into the blood circulation [83], the beneficial molecules from probiotics in the intestines are also possibly transferred into the circulatory system [52]. Due to the reduced serum cytokines with the presence of LGG in the intestine, LGG might secrete some molecules that might be small enough to pass through the damaged gut barrier during sepsis. Previous studies showed that LGG could reduce damage to intestinal epithelial cells caused by cytokine stimulation and decreased cell apoptosis [71], possibly through the p40 protein (a soluble protein) secreted by LGG that stimulates the antiapoptotic PI3K/Akt pathway [72]. Additionally, other LGG-derived soluble factors that can stimulate cytoprotective pathways in intestinal epithelial cells and inhibit cytokine secretion from macrophages are also mentioned [73,74]. The LGG condition media (LCM) was used with LPS stimulation to test this hypothesis. As such, LCM attenuated inflammation in macrophage (RAW 264.7) as indicated by reduced supernatant *TNF-α* and downregulated *IL-1β*, with neutralization of *TLR-4* expression, but no effect on microglia and hippocampal cells. These data implied some anti-inflammatory molecules in LCM against macrophages; however, there was no direct anti-inflammatory impact on microglia and hippocampal cells. Although microglia are mentioned as macrophages, there are several differences between these cells [84], which is out of the scope of the study. Nevertheless, our results implied that the sepsis attenuation of LGG might partly be due to an interference in macrophages, especially the intestinal macrophages, but not a direct induction by LGG. The benefits of neurological symptoms/scores in LGG-administered sepsis mice might be due to an attenuation of systemic inflammation, which indirectly affects the brain. Notably, the additive effect of D-gal on LPS-induced inflammation was demonstrated only in microglia (expression of *TNF-α*, *IL-1β*, and *iNOS*) and in hippocampal cells (supernatant *TNF-α* with expression of *TNF-α*, *IL-6*, and *iNOS*), but not in macrophages (RAW264.7 cells). These data suggest a difference in D-gal metabolism in microglia and hippocampal cells versus RAW264.7 cells, supporting a well-known D-gal as “brain sugar” due to the abundance in nerve tissue [16]. Notably, the selected concentrations of LPS at 100 ng/mL are categorized as a very high concentration of LPS, and the level of endotoxemia during sepsis is usually less than 1 endotoxin unit per mL (EU/ mL) and 0.1 to 0.2 ng/mL is equal to 1 EU/mL [85]. Meanwhile, the selected D-gal concentration at 100 mM is also possibly higher than the level in the mice which depends on the pharmacokinetics of D-gal after subcutaneous injection (120 mg/kg/dose). Although the selected concentrations of LPS and D-gal here might be very high when compared with the concentrations in mice, macrophage activation by both molecules was a proof of concept for the possible additive pro-inflammatory effect between LPS and D-gal. More studies with the proper design experiments are interesting. Moreover, exploration of probiotics in other special mice with aging hallmarks, for example, mice with the deficiency in myelocytomatosis oncogene (*Myc*) [86] and/ or apoptosis signal-regulating kinase 1 (ASK-1) gene [87], might be fascinating to determine more details on the mechanisms of probiotics on sepsis attenuation.

## Conclusions

In conclusion, the gut microbiome composition of D-gal-induced aging mice was different from the control mice, especially the reduced Firmicutes with elevated Bacteroidota, and LGG administration attenuated aging-induced gut dysbiosis with enhanced some beneficial bacteria (Akkermansiaceae). Also, LGG might produce some anti-inflammatory molecules that directly attenuate enterocyte inflammation, resulting in an indirectly improved sepsis condition. Our results support using probiotics as an adjuvant treatment for severe sepsis. More studies are interesting.

## Supporting information

S1 Table Statistical summarization. (DOCX)
