# Supplementary material for: Aging-induced dysbiosis worsens sepsis severity but is attenuated by probiotics in D-galactose-administered mice with cecal ligation and puncture model
Source: PLoS One. 2024 Oct 18;19(10):e0311774. doi: 10.1371/journal.pone.0311774 (PMC11488720; doi:10.1371/journal.pone.0311774)
Supplement: S1 Table — (DOCX) [file pone.0311774.s001.docx]

1. **Survival (Log-rank test)**

|  | Cont_PBS | LGG_PBS | Cont_D-gal | LGG_D-gal |
| --- | --- | --- | --- | --- |
| Cont_PBS | non-sig | 0.021 | non-sig | non-sig |
| LGG_PBS | 0.021 | non-sig | non-sig | 0.038 |
| Cont_D-gal | non-sig | 0.030 | non-sig | non-sig |
| LGG_D-gal | non-sig | non-sig | non-sig | non-sig |

1. **Serum creatinine (ANOVA with Tukey analysis)**

|  | Cont_Sham | LGG_Sham | Cont_CLP | LGG_CLP | (D-gal)  Cont_Sham | (D-gal)  LGG_Sham | (D-gal)  Cont_CLP | (D-gal)  LGG_CLP |
| --- | --- | --- | --- | --- | --- | --- | --- | --- |
| Cont_Sham | non-sig | non-sig | 0.001 | 0.001 | non-sig | non-sig | 0.002 | 0.004 |
| LGG_Sham | non-sig | non-sig | 0.001 | 0.001 | non-sig | non-sig | 0.002 | 0.004 |
| Cont_CLP | 0.001 | 0.001 | non-sig | 0.01 | <0.001 | <0.001 | 0.023 | non-sig |
| LGG_CLP | 0.001 | 0.001 | 0.010 | non-sig | <0.001 | <0.001 | 0.001 | 0.020 |
| (D-gal)  Cont_Sham | non-sig | non-sig | <0.001 | <0.001 | non-sig | non-sig | <0.001 | 0.002 |
| (D-gal)  LGG_Sham | non-sig | non-sig | <0.001 | <0.001 | non-sig | non-sig | <0.001 | 0.002 |
| (D-gal)  Cont_CLP | 0.002 | 0.002 | 0.023 | 0.001 | 0.001 | 0.001 | non-sig | 0.045 |
| (D-gal)  LGG_CLP | 0.005 | 0.004 | non-sig | 0.020 | 0.002 | 0.002 | 0.044 | non-sig |

1. **Alanine transaminase (ANOVA with Tukey analysis)**

|  | Cont_Sham | LGG_Sham | Cont_CLP | LGG_CLP | (D-gal)  Cont_Sham | (D-gal)  LGG_Sham | (D-gal)  Cont_CLP | (D-gal)  LGG_CLP |
| --- | --- | --- | --- | --- | --- | --- | --- | --- |
| Cont_Sham | non-sig | non-sig | <0.001 | 0.005 | non-sig | non-sig | <0.001 | <0.001 |
| LGG_Sham | non-sig | non-sig | <0.001 | <0.001 | non-sig | non-sig | <0.001 | <0.001 |
| Cont_CLP | <0.001 | <0.001 | non-sig | 0.015 | <0.001 | 0.001 | 0.008 | non-sig |
| LGG_CLP | 0.005 | <0.001 | 0.014 | non-sig | 0.003 | 0.013 | <0.001 | 0.047 |
| (D-gal)  Cont_Sham | non-sig | non-sig | <0.001 | 0.003 | non-sig | non-sig | <0.001 | <0.001 |
| (D-gal)  LGG_Sham | non-sig | non-sig | 0.001 | 0.013 | non-sig | non-sig | <0.001 | <0.001 |
| (D-gal)  Cont_CLP | <0.001 | <0.001 | 0.008 | <0.001 | <0.001 | <0.001 | non-sig | 0.012 |
| (D-gal)  LGG_CLP | <0.001 | <0.001 | non-sig | 0.044 | 0.044 | <0.001 | 0.012 | non-sig |

1. **SHIRPA Score (ANOVA with Tukey analysis)**

|  | Cont_Sham | LGG_Sham | Cont_CLP | LGG_CLP | (D-gal)  Cont_Sham | (D-gal)  LGG_Sham | (D-gal)  Cont_CLP | (D-gal)  LGG_CLP |
| --- | --- | --- | --- | --- | --- | --- | --- | --- |
| Cont_Sham | non-sig | non-sig | <0.001 | 0.011 | non-sig | non-sig | <0.001 | 0.002 |
| LGG_Sham | non-sig | non-sig | <0.001 | 0.011 | non-sig | non-sig | <0.001 | 0.002 |
| Cont_CLP | <0.001 | Sig. (p<0.05) | non-sig | 0.047 | <0.001 | <0.001 | non-sig | 0.025 |
| LGG_CLP | 0.011 | 0.011 | 0.047 | non-sig | 0.004 | 0.004 | non-sig | non-sig |
| (D-gal)  Cont_Sham | non-sig | non-sig | <0.001 | 0.004 | non-sig | non-sig | <0.001 | 0.001 |
| (D-gal)  LGG_Sham | non-sig | non-sig | <0.001 | 0.004 | non-sig | non-sig | <0.001 | 0.001 |
| (D-gal)  Cont_CLP | <0.001 | <0.001 | non-sig | <0.001 | <0.001 | <0.001 | non-sig | 0.035 |
| (D-gal)  LGG_CLP | 0.002 | 0.002 | 0.025 | non-sig | 0.001 | 0.001 | 0.035 | non-sig |

1. **Serum IL-6 (ANOVA with Tukey analysis)**

|  | Cont_Sham | LGG_Sham | Cont_CLP | LGG_CLP | (D-gal)  Cont_Sham | (D-gal)  LGG_Sham | (D-gal)  Cont_CLP | (D-gal)  LGG_CLP |
| --- | --- | --- | --- | --- | --- | --- | --- | --- |
| Cont_Sham | non-sig | non-sig | <0.001 | 0.002 | non-sig | non-sig | <0.001 | <0.001 |
| LGG_Sham | non-sig | non-sig | <0.001 | 0.002 | non-sig | non-sig | <0.001 | <0.001 |
| Cont_CLP | <0.001 | <0.001 | non-sig | 0.002 | <0.001 | <0.001 | non-sig | non-sig |
| LGG_CLP | 0.002 | 0.002 | 0.002 | non-sig | 0.001 | 0.001 | 0.026 | 0.040 |
| (D-gal)  Cont_Sham | non-sig | non-sig | <0.001 | 0.001 | non-sig | non-sig | <0.001 | <0.001 |
| (D-gal)  LGG_Sham | non-sig | non-sig | <0.001 | 0.001 | non-sig | non-sig | <0.001 | <0.001 |
| (D-gal)  Cont_CLP | <0.001 | <0.001 | non-sig | 0.026 | <0.001 | <0.001 | non-sig | non-sig |
| (D-gal)  LGG_CLP | <0.001 | <0.001 | non-sig | 0.040 | <0.001 | <0.001 | non-sig | non-sig |

1. **Serum TNF-α (ANOVA with Tukey analysis)**

|  | Cont_Sham | LGG_Sham | Cont_CLP | LGG_CLP | (D-gal)  Cont_Sham | (D-gal)  LGG_Sham | (D-gal)  Cont_CLP | (D-gal)  LGG_CLP |
| --- | --- | --- | --- | --- | --- | --- | --- | --- |
| Cont_Sham | non-sig | non-sig | <0.001 | <0.001 | non-sig | non-sig | 0.001 | 0.024 |
| LGG_Sham | non-sig | non-sig | <0.001 | <0.001 | non-sig | non-sig | 0.001 | 0.020 |
| Cont_CLP | <0.001 | <0.001 | non-sig | 0.006 | <0.001 | <0.001 | non-sig | 0.024 |
| LGG_CLP | <0.001 | <0.001 | 0.006 | non-sig | <0.001 | <0.001 | 0.030 | non-sig |
| (D-gal)  Cont_Sham | non-sig | non-sig | <0.001 | <0.001 | non-sig | non-sig | 0.001 | 0.019 |
| (D-gal)  LGG_Sham | non-sig | non-sig | <0.001 | <0.001 | non-sig | non-sig | 0.001 | 0.019 |
| (D-gal)  Cont_CLP | 0.001 | 0.001 | non-sig | 0.030 | 0.001 | 0.001 | non-sig | 0.001 |
| (D-gal)  LGG_CLP | 0.024 | 0.020 | 0.024 | non-sig | 0.019 | 0.019 | 0.001 | non-sig |

1. **Serum IL-1ß (ANOVA with Tukey analysis)**

|  | Cont_Sham | LGG_Sham | Cont_CLP | LGG_CLP | (D-gal)  Cont_Sham | (D-gal)  LGG_Sham | (D-gal)  Cont_CLP | (D-gal)  LGG_CLP |
| --- | --- | --- | --- | --- | --- | --- | --- | --- |
| Cont_Sham | non-sig | non-sig | <0.001 | <0.001 | non-sig | non-sig | <0.001 | 0.008 |
| LGG_Sham | non-sig | non-sig | <0.001 | <0.001 | non-sig | non-sig | <0.001 | 0.007 |
| Cont_CLP | <0.001 | <0.001 | non-sig | 0.001 | <0.001 | <0.001 | non-sig | 0.006 |
| LGG_CLP | <0.001 | <0.001 | 0.001 | non-sig | <0.001 | <0.001 | <0.001 | non-sig |
| (D-gal)  Cont_Sham | non-sig | non-sig | <0.001 | <0.001 | non-sig | non-sig | <0.001 | 0.007 |
| (D-gal)  LGG_Sham | non-sig | non-sig | <0.001 | <0.001 | non-sig | non-sig | <0.001 | 0.007 |
| (D-gal)  Cont_CLP | <0.001 | <0.001 | non-sig | <0.001 | <0.001 | <0.001 | non-sig | 0.001 |
| (D-gal)  LGG_CLP | 0.008 | 0.007 | 0.006 | non-sig | 0.007 | 0.007 | 0.001 | non-sig |

1. **Serum IL-10 (ANOVA with Tukey analysis)**

|  | Cont_Sham | LGG_Sham | Cont_CLP | LGG_CLP | (D-gal)  Cont_Sham | (D-gal)  LGG_Sham | (D-gal)  Cont_CLP | (D-gal)  LGG_CLP |
| --- | --- | --- | --- | --- | --- | --- | --- | --- |
| Cont_Sham | non-sig | non-sig | <0.001 | 0.011 | non-sig | non-sig | <0.001 | 0.011 |
| LGG_Sham | non-sig | non-sig | 0.010 | 0.011 | non-sig | non-sig | <0.001 | 0.011 |
| Cont_CLP | <0.001 | 0.010 | non-sig | 0.021 | <0.001 | <0.001 | non-sig | 0.038 |
| LGG_CLP | 0.011 | 0.011 | 0.021 | non-sig | 0.011 | 0.011 | 0.038 | non-sig |
| (D-gal)  Cont_Sham | non-sig | non-sig | <0.001 | 0.011 | non-sig | non-sig | <0.001 | 0.011 |
| (D-gal)  LGG_Sham | non-sig | non-sig | <0.001 | 0.011 | non-sig | non-sig | <0.001 | 0.011 |
| (D-gal)  Cont_CLP | <0.001 | <0.001 | non-sig | 0.038 | <0.001 | <0.001 | non-sig | 0.021 |
| (D-gal)  LGG_CLP | 0.011 | 0.011 | 0.038 | non-sig | 0.011 | 0.011 | 0.021 | non-sig |
